# Novel assessment of risk tolerance in acute healthcare settings: a questionnaire-based study investigating risk tolerance of service users and staff in ambulatory care and front-door services

**DOI:** 10.1136/bmjopen-2025-099032

**Published:** 2025-11-12

**Authors:** Ciara Harris, Johannes Lohse, Michalis Drouvelis, Daniel S Lasserson

**Affiliations:** 1 Warwick Applied Health, University of Warwick Medical School, Coventry, UK; 2 Institute of Applied Health Research, University of Birmingham College of Medical and Dental Sciences, Birmingham, UK; 3 Department of Economics, University of Birmingham, Birmingham, UK; 4 Institute of Economics, Leuphana University Lüneburg School of Business and Economics, Lüneburg, Germany; 5 CESifo GmbH, Munich, Germany; 6 Department of Acute Medicine, University Hospitals Coventry and Warwickshire NHS Trust, Coventry, UK; 7 Acute Hospital at Home, Department of Geratology, Oxford University Hospitals NHS Foundation Trust, Oxford, UK

**Keywords:** Decision Making, Behavior, GENERAL MEDICINE (see Internal Medicine), Patients

## Abstract

**Abstract:**

**Objectives:**

When deciding acute healthcare delivery location, multiple factors should be considered, including risks associated with potential care locations and the willingness of decision stakeholders to take those risks. Individual risk tolerance potentially informs these choices. We therefore aimed to investigate the risk tolerance of staff, patients and carers in front-door and ambulatory care units.

**Design:**

Several variants of the ‘multiple price list’ method of risk tolerance assessment were employed. The different variants covered financial and health outcomes, and known and unknown odds in the ‘risky’ options. For financial outcomes, participants made seven choices between a guaranteed (eg, £70) and risky (eg, chance of £20 or £160) outcome, with the higher quantity in the risky outcome increasing with each choice, in six ‘lottery sets’. For health outcomes, participants made choices between a guaranteed and risky outcome measured in number of healthy days.

**Setting and participants:**

Staff, patients and carers were recruited from front-door and ambulatory care units in the UK.

**Outcome measures:**

Risk tolerance was the primary outcome measure and was established in two ways—number of times the guaranteed option was chosen, and the point where participants switched from the guaranteed to the risky option.

**Results:**

Among 338 participants, a wide range of risk tolerance levels were demonstrated, and three key findings were identified—participants were less risk tolerant in health-based than financial decisions; older people had a more dichotomised approach to health risk-taking than younger people; and patients could engage in informed, structured discussions about risk, including when acutely unwell.

**Conclusions:**

These findings suggest that, while stakeholders in location-of-care decisions may have different risk tolerance levels, they can engage in structured discussions about risk, which should inform shared decision-making. Additionally, older patients, who constitute a significant proportion of hospital attendees, may be more willing to take health-based risks than younger people. Future work may benefit from formal exploration of people’s rationale for their decisions and may be considered in other clinical settings.

**STRENGTHS AND LIMITATIONS OF THIS STUDY**

- This study represents the first time, to the authors’ knowledge, this formalised approach has been taken to analysing risk tolerance in an acute care setting, using the same approach for staff, patients and carers.
- This study engaged patients who were acutely unwell in structured discussions about risk, which has not previously been done in this way in a research study.
- Multiple approaches were taken to facilitate patient participation, allowing those with low English literacy, numeracy and/or digital skills to participate.
- Informal field notes were used to provide additional context to some of the choices that participants made, which has not been used as an approach in previous work of this type and therefore contributed to providing insights into the decision-making process that would have been otherwise missed.
- Limitations of this study included the lower proportion of older people who participated, compared with the proportion of people presenting for unplanned care who are older, and the under-representation of some professionals in the staff cohort, such as nurses, although doctors and physiotherapists were both well-represented.

## Introduction

 When patients present with acute health decline requiring hospital-level care, a decision must be made about where their care is provided. There are various options available, including ambulatory care (where the patient attends regularly for treatment but returns to their own home overnight^1^), Hospital at Home (HaH) (where the patient remains at home and hospital-level care is brought to them^2^) and admission to an inpatient ward. All of these care locations carry idiosyncratic risks, such as the risk of deconditioning^3^ or delirium^4^ as an inpatient, and concerns about unobserved deterioration^5^ and data security^6^ in a non-inpatient setting. Risks such as these should be accounted for when making the location of care decision. Given the importance of involving patients in decisions about their own care,^7 8^ it is important to ensure that they are informed about these risks, such that this information can be factored into their decision-making about whether they wish to take certain risks, in line with their risk tolerance or preference, as well as that of the health professional, when making location of care decisions at the hospital front-door. Additionally, patients’ families or carers are also important stakeholders in location of care decisions, and when they are involved their risk tolerance should also be considered. To date, there have been few investigations into understanding the risk preferences of patients within an acute care setting. Therefore, there is little knowledge on the appropriate methods to assess risk preferences in this particular setting.

Previous investigations of risk tolerance, or preference, have rarely included healthcare settings, or patients, and none have investigated this within acute care settings. Much of the previous work in this field has instead focused on selected populations, typically drawn from convenience samples of university students, although notable exceptions to this include Anderson *et al* ^9^ and Falk *et al*,^10^ who have studied a broader population of subjects, yet not in an acute care setting. Previous work conducted in a healthcare setting, and including patients, has been undertaken in outpatient departments,^11^,^13^ and the first two of these studies took place in Greece during the financial crash in the early 2010s. University students are not necessarily representative of the broader population who work in or attend acute care, more abstract tasks have been used for some of these studies and, to the authors’ knowledge, no studies using these types of approaches have been conducted in a setting where patients are experiencing acute health decline, or within the UK National Health Service (NHS). We, therefore, sought to include participants with a wider range of backgrounds. Within this population, we are assessing whether a standard task previously used in laboratory and online investigations (a variation of the ‘multiple price list’ design^14^) can be adopted and applied in the context of an acute care setting.

Most previous investigations of risk tolerance in behavioural economics have used financial choices as the basis for assessment of risk tolerance^14^,^16^, and therefore this is the setting in which most risk elicitation methods have been validated. The basic finding from this literature and corresponding theories is that most people are moderately risk averse for the tasks and stakes used. However, there is some discussion in the literature to what degree risk preferences are domain specific.^17^

In particular, there is some evidence that people treat financial and health-related risks differently.^11 13^ For the purpose of this study, it is therefore logical to investigate both aspects of risk taking and their relationship, in order to determine if financial risk tolerance is a suitable proxy for health risk tolerance. Additionally, previous research involving patients has found that participants found it easier to understand health-related risk questions if they were presented with similar finance-related questions first,^11^ and therefore this approach was also taken in this study.

The main aim of this study was to establish the feasibility of using multiple price list design lotteries with both financial and health outcomes, to investigate the risk tolerance of healthcare staff, patients and carers in an acute medical setting. Hence, this study is intended as a guide that introduces a research design borrowed from behavioural economics to practitioners and researchers in the area of medical sciences, and in particular in acute medical care.

## Methods

### Participants and recruitment

Staff participants were primarily recruited online, via routes such as Twitter and emails from relevant professional groups. Emails and social media posts included brief information about the study and eligibility criteria, alongside access to a full participant information sheet and a link to participate in the study. Participants were also encouraged to share information about the study with colleagues, and those working in the department where patients and carers were recruited were invited to participate in person. Staff were recruited between August and December 2020. Patients and carers were recruited in person, while they were waiting in an ambulatory care unit of a busy city hospital in Birmingham, UK, between September and December 2020. To be eligible, staff participants had to be a qualified healthcare professional, currently working in an ambulatory care, front-door or HaH service and contribute to location of care decisions. There were no exclusions on the basis of profession, and participants did not have to work exclusively in the relevant unit, provided that at least part of their role involved contributing to location of care decisions there. Patients and carers had to be aged 16 years or older, be a current patient (or carer of a current patient) in an ambulatory care or front-door unit, be able to understand written or spoken English and have capacity to provide informed consent.

Limited previous research of this type in a healthcare setting meant that an ex-ante sample size calculation based on statistical-power considerations was not possible, and target sample size was therefore based on the target number of participants in a previous outpatient-based study involving patients and doctors.^12^ A convenience sampling approach was used for patients and carers, through inviting those who attended the ambulatory care unit during the study period. A combination of convenience and snowball sampling was used for staff participants, through approaching relevant individuals through professional societies and social media, where eligible individuals were likely to see the invitation and encouraging participants to share information about the study with colleagues.

Staff participants completed the study questionnaire independently online, in their own time, but had contact details for the research team, should they require support or to ask any questions. Patients and carers were given a choice about how to complete the study questionnaire—using a laptop independently, the researcher managing the laptop while the participant read the questions and directed all actions, or the researcher verbally asking all questions and managing the laptop.

### Questionnaire

**Figure 1:**
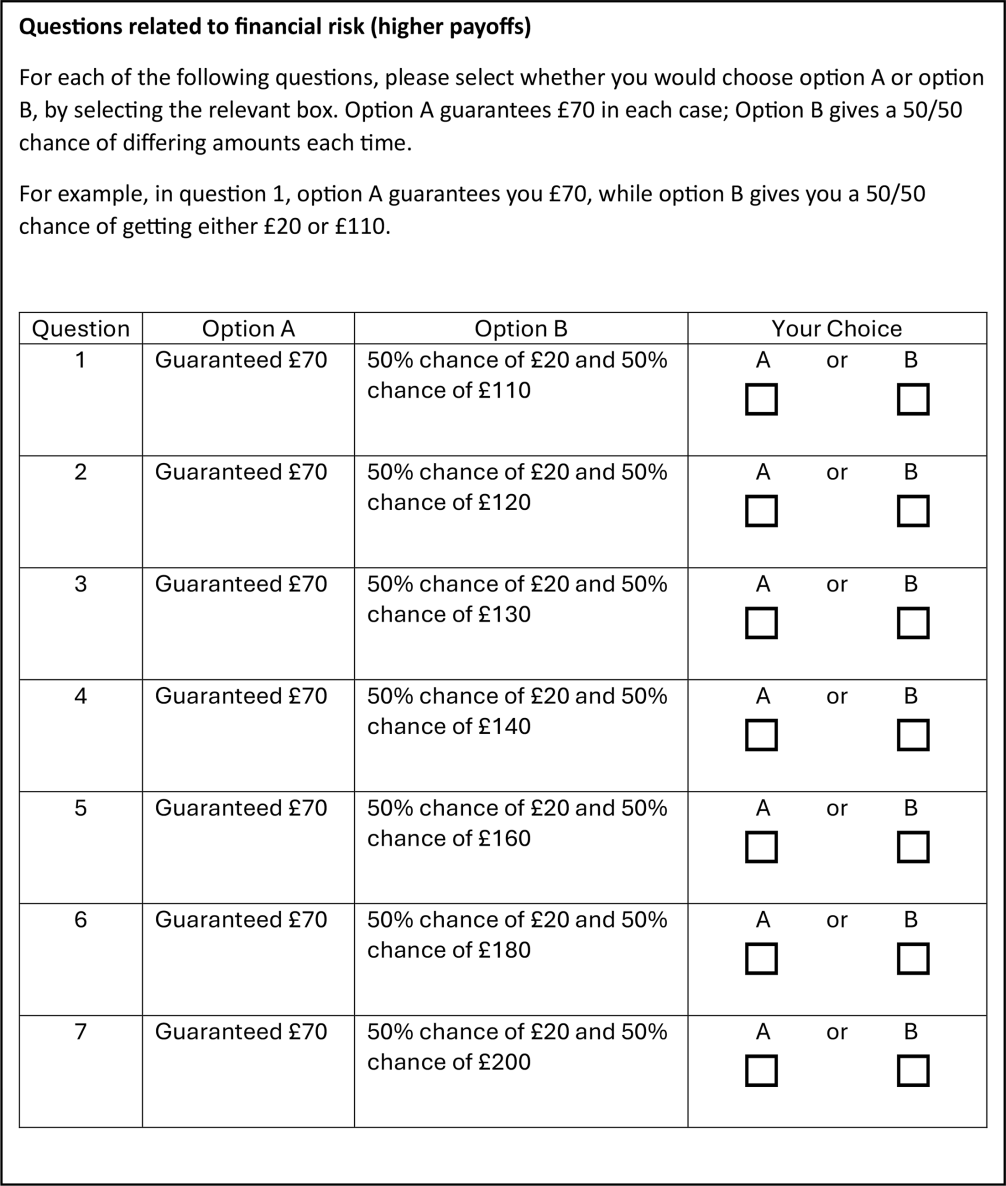
Example of financial lottery set (higher financial payoffs, known chances in option B).

The study questionnaire comprised a set of demographic questions (onlinesupplemental figures 1a  1b, for patients/carers and staff, respectively), followed by a series of six lottery sets (online supplemental figure 2), incorporating financial and health decisions, with different levels of ambiguity in the chances of given outcomes across the six sets. The questionnaire was adapted from previous research which developed and validated a variety of tools to assess aspects of financial risk preferences,^16^ and adaptations were made for this study based on pilot feedback, to increase ease of understanding. What differed between the financial and health lottery questions was the type of outcomes. For financial lotteries, outcomes were expressed in terms of financial payments. For health lotteries, outcomes were expressed in terms of the number of healthy days. Each of the lottery sets included seven ‘lottery questions’ where the participant was asked to choose between a certain payoff (option A) and an uncertain payoff (option B), where the outcome may have been higher or lower than the certain payoff. Across the seven lottery questions, the higher amount in the uncertain payoff increased while the lower payoff remained the same, thus theoretically making the uncertain payoff increasingly desirable by offering a higher expected value (EV) (example in figure 1). Among the six lottery sets, four presented financial choices and two presented health choices. In half of these (two financial and one health lottery set) the chances of each outcome in option B (uncertain payoff) were unknown, representing choices with uncertainty, and in the subsequent half the chances for option B were known to be 50/50, representing choices about risk. This allowed investigation of risk (ambiguity) tolerance in situations where risk or uncertainty levels were framed in financial or health terms. Recruiting different participant groups to take all six lottery sets allowed for comparison between staff and service user groups in both types of situation.

The four financial lottery sets involved both low (option A = guaranteed £7, option B =£2 or between £11 and £20) and high (quantities increased by a factor of 10, as in figure 1) financial stakes. The first time each of these was presented to participants, the probabilities in the uncertain option B were unknown (online supplemental figure 2), and the second time they were presented (after the first health domain lottery set), the probabilities in the second option (B) were stated as 50/50. The versions with unknown probabilities were presented before those with known probabilities to reduce the risk of participants assuming the same probabilities across both types of lottery set.

A similar approach was taken with the health domain lottery sets, with ‘days in full health’ replacing the financial payoffs, and the number of ‘days in full health’ matching the higher financial quantities (Treatment Option A = 70 days in full health, Treatment Option B = 20 days or between 110 and 200 days in full health; online supplemental figure 2). For the health lottery sets, participants were asked to imagine that they had a health condition which prevented them from completing their normal daily activities, such as hobbies or work, and that they were being presented with two treatment options, which offered differing periods of time ‘in full health’. As with the financial lottery sets, the set with unknown probabilities (Treatment Option B) in the uncertain option was presented first, such that all lottery sets with unknown probabilities were presented prior to all those with known probabilities.

### Determination of risk tolerance and management of unusual choice patterns

Across the six lottery sets (with seven decisions each) participants hence choose 42 times between a safer and a riskier option. As is common in the literature, risk tolerance can be determined based on these choices in two equally valid ways—the number of times a participant chose the guaranteed option (referred to as ‘number of A’s’), and the point at which a participant switched from the guaranteed to the uncertain option for the first time (referred to as ‘Switch Point’). In both cases, risk preference was initially determined for each lottery set separately. Some subsequent analyses combined scores across multiple lottery sets where relevant (eg, to compare health lottery sets with financial ones).

Existing studies on risk preferences suggest that the most common behavioural pattern in these lottery sets is for a participant to initially choose the guaranteed option and at some point switch to the uncertain/risky option.^9^ It may also be consistent with standard assumptions about risk preferences for a participant to not switch at any point, and instead to choose the guaranteed or the uncertain option for all choices in the lottery set. The former would indicate very high levels of risk aversion while the latter would indicate high levels of risk tolerance (risk loving preferences). These empirical patterns can be interpreted via standard theories of decision-making under risk. In an expected utility framework, for instance, risk-neutral decision-makers would accept a lottery if its EV is at least as high as the guaranteed outcome. Choosing the risky option when its EV is lower than the guaranteed outcome indicates risk-loving preferences, whereas switching to the risky option, only when its EV is higher than the guaranteed outcome indicates risk aversion.^18^ In non-expected utility frameworks, such as prospect theory,^19^ decision-makers may additionally compare outcomes to a reference point and subjectively weight probabilities.

Despite these unique switching patterns being the three most common patterns in multiple price lists, alternative choice patterns may also occur, for various reasons. The most frequent atypical patterns would be to switch multiple times between the guaranteed and uncertain options within a single lottery set (referred to as ‘multiple switchers’) or to initially choose the uncertain option and later switch to the guaranteed option. These choice patterns were managed slightly differently during analyses.

Those with either of the first two choice patterns (switching once from the guaranteed to the uncertain option, or not switching at all) were included in all analyses, and both methods of determining risk tolerance were used. The Switch Point for those who switched once matched the point at which they began selecting the uncertain option (eg, a participant who chose the uncertain option from lottery question 4 onwards had a Switch Point of 4). For those who did not switch, their Switch Point was deemed to be 8 if they chose the guaranteed option every time (ie, one higher than switching on the last lottery question, demonstrating high risk aversion) and one if they always chose the uncertain option (ie, the equivalent of switching to the uncertain option on the first question, demonstrating high risk tolerance).

The data from ‘multiple switchers’ were included in the analyses using the number of times the guaranteed option was chosen, as this could still be accurately calculated without those choices of the guaranteed option needing to be consecutive. However, due to lacking a single Switch Point, it was unclear how to interpret the risk tolerance of those with multiple Switch Points, and they were therefore excluded from Switch Point analyses. For those who switched multiple times in only some lottery sets, their data were excluded only from those lottery sets in which they switched multiple times. Finally, those with a single Switch Point from the uncertain to guaranteed option were included in the ‘number of A’s’ analyses, and for the ‘Switch Point’ method, analyses were conducted with and without these participants, using the point when they switched from the uncertain option to the guaranteed one as their Switch Point.

### Statistical analysis

Statistical analyses were conducted using IBM SPSS Statistics V.27 software, and included both whole population analyses and subgroup analyses. The subgroup analyses comprised comparisons between groups with different demographic characteristics, as well as comparisons of risk tolerance between types of lottery set (eg, health vs financial, ambiguous vs known probabilities). Risk tolerance scores overall, for health, for finance, with ambiguity and without ambiguity were calculated separately.

Descriptive statistics and histograms were used to demonstrate patterns of risk tolerance, for both whole population and subgroup analyses. Averages for both ‘number of A’s’ and Switch Point analyses are presented as mean (SD) or median (IQR), dependent on the distribution of the data. The Mann-Whitney U test was used to assess between-group differences.

Different types of regression analyses were used for the two different methods of determining risk tolerance. Multiple regression analyses were conducted for the method using the number of times the guaranteed option was chosen, and ordinal logistic regression analyses were conducted for the Switch Point method (including only those who switched once or did not switch). Due to the difference in independent variables, these regression analyses were conducted separately for staff and patients and carers.

## Results

Between August and December 2020, 338 participants were recruited and completed the questionnaire (106 healthcare professionals, 197 patients and 35 carers). Participants’ demographics are shown in tables 1a and 1b, with more female than male participants overall (63.3% female). Among healthcare professionals, multiple professions were represented, with doctors and physiotherapists being the most common, and they had a range of experience levels (table 1a). Patient and carer participants were aged from 16 to over 85 years old and had a range of ethnicities (table 1b). An additional cut-off point of age categorisation was added at 64 years, in order to allow analyses based on those aged less than 65 years, and those aged 65 years and older, in line with common practice of categorising 65 years as the minimum age for ‘older people’.^20^ Notably, the timing of recruitment for this study meant that patients and carers were recruited from a hospital setting during the COVID-19 pandemic, which reduced the number of carers who were present in the department to be approached about participation. Staff were recruited primarily online, and therefore this may have had less of an impact on their recruitment, although increased and changing workloads as a result of the pandemic may also have impacted recruitment. Reasons for non-participation were not formally recorded, but 67.7% of patients and carers approached about participation consented to participate.

**Table 1:** (a) Demographics of healthcare professionals. (b) Patient and Carer demographics

Characteristic	All staff (n=106)
**a**	
Gender* (%) female	62 (58.5)
Job role (%)	Doctor: 67 (63.2)
	Physio: 26 (24.5)
	OT: 4 (3.8)
	Nurse: 7 (6.6)
	Other: 2 (1.9)
Years working since qualification (%)	15+: 54 (50.9)
	11–14: 15 (14.2)
	6–10: 21 (19.8)
	2–5: 13 (12.3)
	Less than 2: 3 (2.8)
Service type—binary (% of binary group)†	Ambulatory care: 21 (30.9)
	Front-door: 47 (69.1)
Service type—not binary (% of non-binary group)‡	Ambulatory care only: 5 (13.2)
	Front-door only: 7 (18.4)
	Both: 26 (68.4)

Characteristic	All patients and carers (n=232)	Patients only (n=197)	Carers only (n=35)
**b**			
Gender* (%) female	152 (65.5)	129 (65.5)	23 (65.7)
Age category (%)	16–20: 14 (6.0)	16–20: 13 (6.6)	16–20: 1 (2.9)
	21–30: 48 (20.7)	21–30: 38 (19.3)	21–30: 10 (28.6)
	31–40: 52 (22.4)	31–40: 49 (24.9)	31–40: 3 (8.6)
	41–50: 34 (14.7)	41–50: 29 (14.7)	41–50: 5 (14.3)
	51–60: 43 (18.5)	51–60: 35 (17.8)	51–60: 8 (22.9)
	61–64: 8 (3.4)	61–64: 6 (3.0)	61–64: 2(5.7)
	65–74: 18 (7.8)	65–74: 14 (7.1)	65–74: 4 (11.4)
	75–84: 14 (6.0)	75–84: 12 (6.1)	75–84: 2 (5.7)
	85–94: 1 (0.4)	85–94: 1 (0.5)	85–94: 0
	95+: 0	95+: 0	95+: 0
Ethnicity (%)	All white: 112 (48.3)	All white: 98 (49.7)	All white: 14 (40)
	All mixed/multiple ethnic groups: 5 (2.2)	All mixed/multiple ethnic groups: 5 (2.5)	All mixed/multiple ethnic groups: 0
	All Asian/Asian British: 63 (27.2)	All Asian/Asian British: 47 (23.9)	All Asian/Asian British: 16 (45.8)
	All Black/African/Caribbean/Black British: 43 (18.5)	All Black/African/Caribbean/Black British: 39 (19.8)	All Black/African/Caribbean/Black British: 4 (11.4)
	All other ethnic groups: 3 (1.3)	All other ethnic groups: 2 (1.0)	All other ethnic groups: 1 (2.9)
	Prefer not to say: 6 (2.6)	Prefer not to say: 6 (3.0)	Prefer not to say: 0
Method of questionnaire completion (%)	Laptop: 43 (18.5)		
	Verbal: 180 (77.6)		
	No data: 9 (3.9)		

* All participants reported their gender as either female or male.

† Participants who were asked their service type as a binary question (n=68).

‡ Participants who could choose one or both service types as the type of service they worked in (n=38).

### Key findings

**Figure 2:**
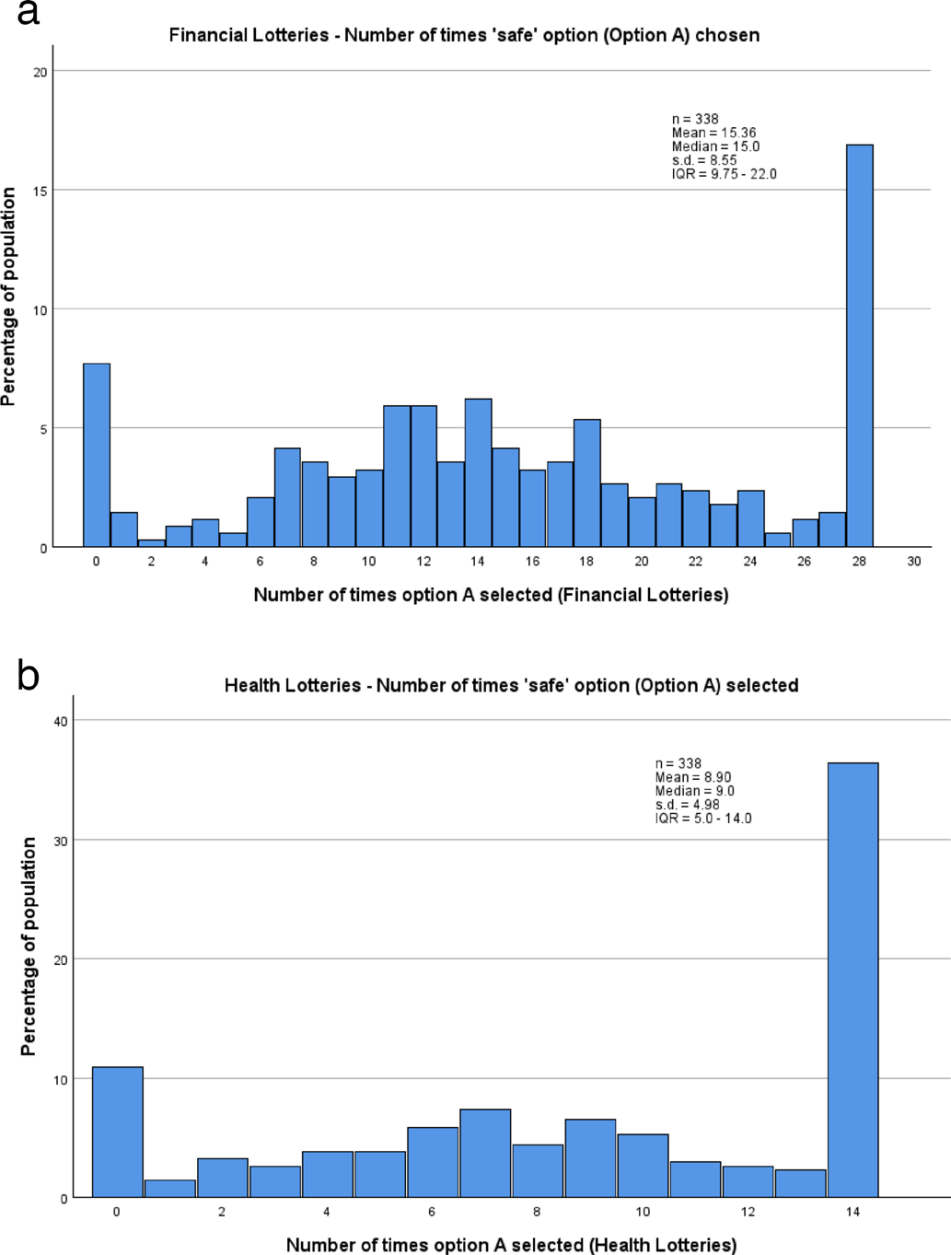
(**a**) Distribution, by percentage, of number of times the ‘safe’ option was chosen by participants, across the four financial lottery sets. (**b**) Distribution, by percentage, of number of times the ‘safe’ option was chosen by participants, across the two health lottery sets.

Overall, a wide range of risk tolerance levels were observed, ranging from those who chose the uncertain option every time (very risk tolerant) to those who chose the guaranteed option every time (very risk averse). This latter pattern was the most common, across the whole population in all six lottery sets and in the financial and health lottery set groups separately (figure 2a,b). When all six lottery sets are combined, that is, initially ignoring any differences between lottery sets, participants make a total of 42 (6 sets×7 choices) choices between a safer and a more risky option. For the four financial lottery sets there are hence 28 choices (4×7).

Among the comparisons between lottery types, the most notable finding was that participants tended to be more risk averse when making choices regarding their health than when making financial decisions. Since there were four financial lottery sets (comprising 28 choices) and only 2 health lottery sets (14 choices), medians for the number of times the guaranteed option was chosen were compared between the two health lottery sets and the two high payoff financial lottery sets (14 choices), in which the quantity of pounds was numerically equal to the number of ‘days in full health’. The higher median choice of the guaranteed option in the health lottery sets (9.0, IQR 5.0–14.0) than the high financial ones (7.0, IQR 4.75–12.0) indicates lower risk tolerance in the health-based decisions. This is also demonstrated by a higher proportion of participants choosing the guaranteed option every time in the health-based lottery sets than the financial ones (36.4% vs 21.3%), and a lower proportion of participants in the health-based lottery sets choosing to take the uncertain option every time compared with choices in the financial lottery sets (10.9% vs 12.4%). As with each lottery set individually, the most common choice pattern in both pairs of lottery sets was to choose the guaranteed option every time (figures2b  3).

**Figure 3:**
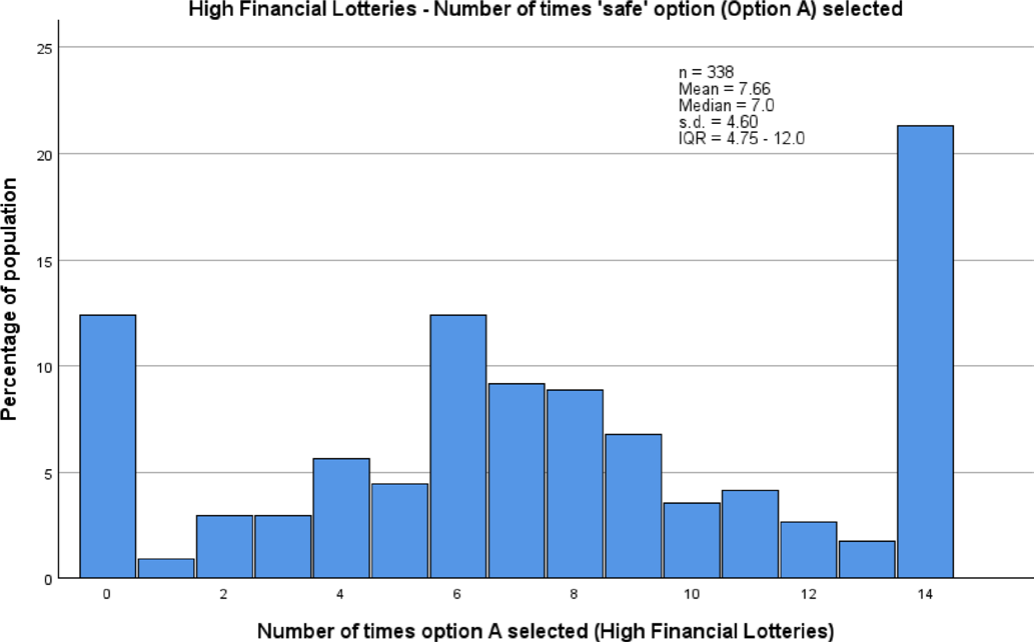
Distribution, by percentage, of number of times the guaranteed option was chosen by participants, across the two high financial payoff lottery sets.

Further supporting this finding, multiple regression, with all lottery sets included, identified statistically significant slope coefficients for lottery type. We find this pattern both among healthcare professionals (−0.492, 95% CI −0.971 to −0.013, p=0.044) and among patients and carers (−0.612, 95% CI −0.922 to −0.302, p<0.001), both indicating that the number of times the guaranteed option was chosen in financial lottery sets was lower than that in health lottery sets, demonstrating greater risk tolerance in the financial sets.

Finally, when the Switch Point was used to identify participants’ risk preferences, a similar finding of greater risk aversion in health lottery sets was identified. Both staff and patients and carers had statistically significant odds of having a higher Switch Point in the health lottery sets than the financial ones (Staff: OR=1.576; 95% CI 1.167 to 2.128; p=0.003. Patients and carers: OR=1.613; 95% CI 1.303 to 1.996; p<0.001). This indicates that they were willing to accept the uncertain option earlier in the financial lottery sets, but continued to choose the guaranteed option for longer in the health lottery sets. Through the lens of expected utility theory, this can be interpreted as choices falling more likely in the risk-averse range of preferences.

**Figure 4:**
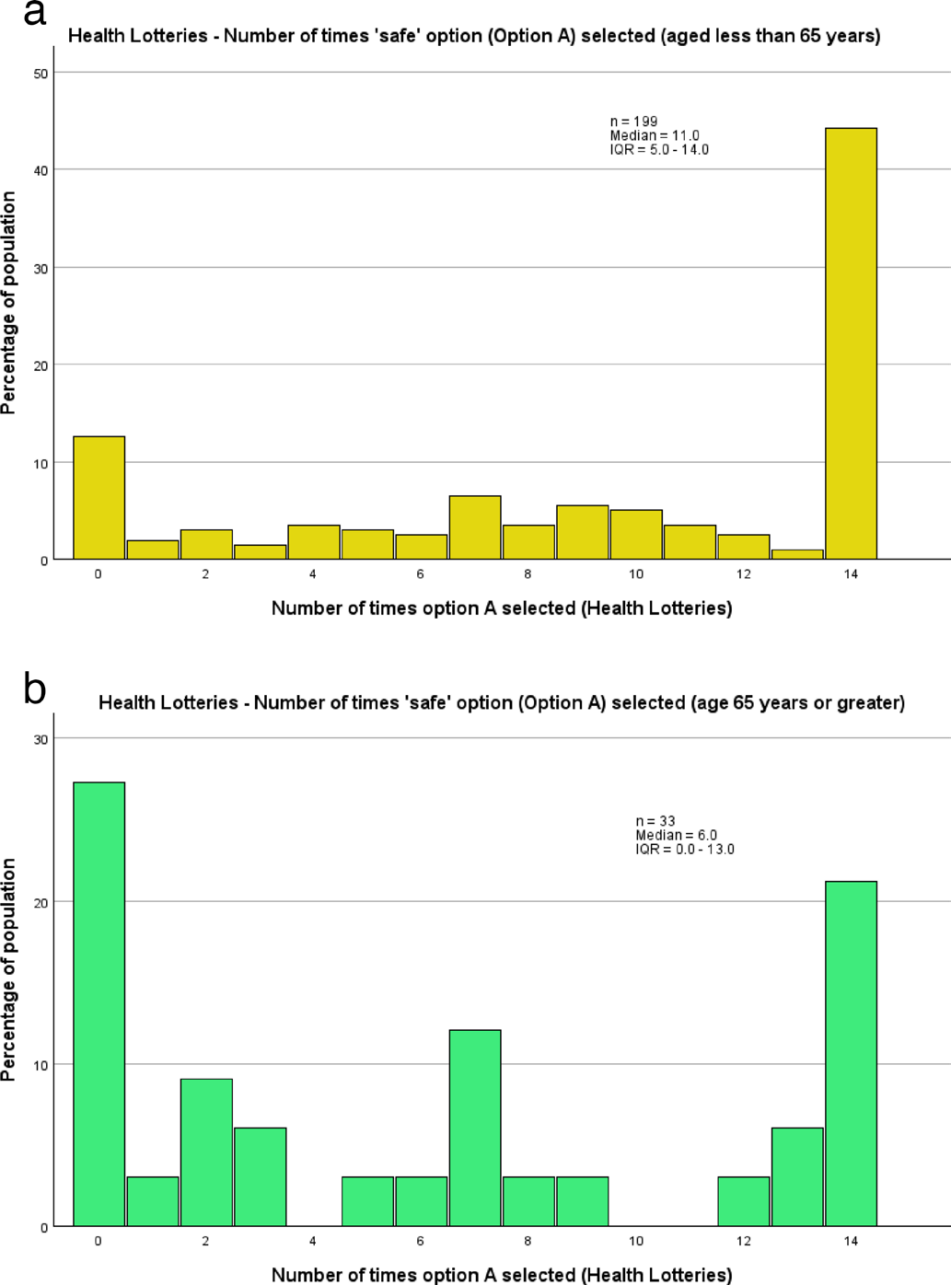
(**a**) Distribution, by percentage, of number of times the ‘safe’ option was chosen by patients and carers aged less than 65 years, across the two health lottery sets. (**b**) Distribution, by percentage, of number of times the ‘safe’ option was chosen by patients and carers aged 65 years or older, across the two health lottery sets.

When comparing between demographic groups, one of the most notable findings was the difference in the choice patterns between older (aged ≥65 years) and younger (aged <65 years) participants, when making health-related choices (14 choices). Older participants tended to have a more dichotomised approach to risk taking in health-based decisions than younger participants. When comparisons were made using the number of times the guaranteed option was chosen, the distributions of these showed a statistically significant difference (p=0.002) between older and younger people, with older people tending to make more choices at the extremes of either high risk tolerance or high risk aversion in the health lottery sets, whereas younger people tended more consistently towards risk aversion (figure 4a,b). Additionally, unlike other subgroups analysed within this study, or the study population overall, the most frequent choice pattern among those aged 65 years or older was to choose the uncertain option for every choice (27.3% of participants aged ≥65 years). People younger than 65 years, however, tended towards the opposite extreme, with 44.2% consistently choosing the guaranteed option. Consequently, older people had a notably lower median number of times the guaranteed option was chosen (6.0, IQR 0.0–13.0), in comparison to younger people (11.0, IQR 5.0–14.0). In the corresponding regression analyses, no statistically significant relationship with age was identified. However, these included age as a continuous variable, as opposed to being dichotomised at age 65, and also included all the lottery sets, as opposed to only the health lottery sets, in which the difference in choice patterns was identified.

Among the remaining demographic factors which were analysed, the most notable finding was the lack of a statistically significant difference in risk tolerance between male and female participants, across all lottery sets, financial lottery sets, health lottery sets and across both methods of determining risk tolerance.

The third key finding of this study was that patients and carers were able to discuss risk, in an informed and structured way, including during an episode of acute ill health, while in an ambulatory care unit and that healthcare professionals were willing and able to engage with a questionnaire of this type. Among the patients and carers, this included participants who were multi-lingual, and those who reported that their English literacy was not adequate to confidently read English, but they were sufficiently confident in spoken English to participate. It also included participants who lacked confidence in their numeracy skills, or reported their numeracy skills to be poor, which was most apparent when presented with the concept of 50/50 chances. This latter challenge was resolved through the use of a more easily understood concept—a coin flip.

## Discussion

This study has demonstrated both the wide range of risk tolerance levels among key stakeholders who need to take part in acute location of care decisions, and that those stakeholders are able to engage in structured discussions and decisions about risk, even when acutely unwell. The combined implication of these two key outcomes is to highlight the importance of including patients and carers, where appropriate, in discussions of risk and not to shy away from including nuance in these discussions, which can be held in a structured way, including concrete discussion of risk levels. This is applicable to a range of healthcare decisions, including around treatment decisions as well as location of care. Additionally, the decisions that patients make should be respected, even if their tolerance of the risk involved in their decision conflicts with the health professional’s tolerance of that risk.

In discussing their choices, a number of patient and carer participants explicitly said that they were treating the decision differently when it related to health, as opposed to finance. Therefore, it may be inferred that the statistical difference identified in this study may be a conscious choice, at least on the part of some people, and greater risk averseness in health decisions compared with financial ones has been identified in a previous study involving patients and healthcare professionals.^11^ This difference in tolerance of risk, consciously or otherwise, is also applicable when considering the difference in approaches taken towards health risk by older people compared with younger people. In particular, it may act as a salient reminder that healthcare professionals should not assume that patients, especially older patients, choosing an objectively more, or less, risky option for their healthcare are misinterpreting the information about their options, or that they are making an unwise or irrational choice. It may be entirely rational to choose the higher-risk option, fully understanding that it is high risk, provided that they have considered the information and are able to weigh this against their own values and priorities.

It is also worth considering that the difference in how risk in health decisions is treated by older and younger people may play out in an age difference between patients and their healthcare providers, who are both important stakeholders in health-based decision-making. A high proportion of patients presenting with acute illness are aged over 65 years,^21^ while only 2% of staff employed in the NHS are aged within this group.^22^ Therefore, it is likely that there will frequently be an age mismatch between the patient and staff member, which may influence their respective tolerance for risks in making healthcare decisions, and it is important that healthcare professionals remain cognisant of the fact that their older patients may be more, or less, inclined to accept risks related to health than they themselves are.

Comparisons with other research investigating risk tolerance in people of different ages are limited, due to the small number of studies that have included participants with the age range included in this study. Much of the previous research has focused on university populations, and as such has not included older people, and most of these studies have not assessed risk tolerance in health-based decisions. Generally, no statistically significant differences based on age have been identified in previous studies investigating risk tolerance in health decisions,^11 13^ and one study which included a nationally representative sample, assessing financial risk tolerance, identified that those aged 40–50 years are more risk tolerant than others, but no other age-related statistically significant differences were reported.^23^

In contrast to consideration of the impact of age, a number of previous studies have included analysis of differences in risk tolerance based on gender, and have found mixed results. A number of these studies have shown greater risk aversion among female participants in risks involving financial gains,^24^,^26^ while others, similarly to the current study, have not found a significant difference based on gender.^11^,^1323^ Interestingly, this gender difference was not found in the studies conducted in a health-based environment, or deliberately involving patients, when decisions were health-based,^11 12^ and even disappeared in health-based decisions in a study where a gendered difference was found in financial decisions.^26^

The fact that multiple participants, with variable barriers to independent completion of the questionnaires, were able to successfully participate in this study, and discuss risk in a structured way, has implications both for discussions of risks between patients, carers and healthcare professionals and for future research around risk tolerance with the general public. It also increases the potential applicability of these results to the population more broadly, compared with previous research using university populations, as it included a more representative sample of the general population. A number of steps were taken to allow participation for those who may not have felt comfortable doing so independently. Chief among these was the option to complete the questionnaires verbally, if a participant wished, which was offered as an option to all patient and carer participants. This addressed a number of potential barriers, without requiring people to indicate that those barriers may exist if they did not wish to—by allowing the questionnaire to be completed verbally, participants who were not functionally literate, or not literate in English, were able to participate without having to read in English and those who were not computer-literate were able to participate without having to use the computer. Additionally, the option to clarify questions meant that analogies could be used where necessary, such as describing a 50/50 chance as being equivalent to a coin toss, which helped to overcome potential numeracy issues. These approaches could be considered both in discussions with patients about risks and decisions, in how conversations and information sharing may be adapted and in future risk tolerance research where this is done with a non-university-based population. However, for them to be most effective, it is probable that they would need to be offered as standard, not to be reliant on the person who needs an adaptation asking for it.

There has been very limited previous research investigating risk tolerance among patients, and in some cases staff, in a healthcare setting using methods similar to those used in this study, and these have involved patients in an outpatient department,^11^,^13^ rather than acutely unwell patients presenting, mainly, for unplanned care. Additionally, where staff were included in these studies,^11 12^ only doctors were recruited, unlike the current study which recruited a variety of healthcare professionals. In these studies, there were some similar findings to the current study, including the tendency towards greater risk aversion in the health domain,^11 12^ although in one such study patients and community members were found to be risk neutral for health-based decisions and more risk averse for financial decisions.^13^ Where previous research has tried to elicit risk preferences in a similar clinical setting to the current study, these have tended to use different methods, such as clinical vignettes,^27^ and as such, direct comparisons between their findings and those of the current study are limited.

Previous studies employing lottery-style questions to elicit risk tolerance, most often in financial contexts, have focused on decision-makers’ aversion to monetary risk. Within the commonly employed framework of expected utility theory, the decision-maker evaluates each option based on its expected utility, that is, the probability-weighted average of the utility derived from all possible monetary payoffs under each option chosen. A commonly assumed functional form is the exponential utility function, $U(x)=\frac{1-e^{-rx}}{r}$ where x denotes the outcome (eg, monetary payments or number of days in full health), and r>0 captures the degree of risk aversion. For a risk-neutral decision-maker (r=0), the utility function is assumed to be linear (U(x)=x), meaning choices depend only on expected values. Risk aversion implies r>0. Rational agents, under this model, should always select the option with the higher expected utility. Accordingly, in the current study, risk-neutral participants would be expected to switch from the certain to the risky option once the expected value of the latter exceeds that of the former (eg, at line three in the questions with known probabilities). Similarly, risk aversion demands a premium in expected value for choosing a risky outcome, while risk-loving decision-makers would even pick a risky option if it offers a lower expected value than the corresponding guaranteed option. Accordingly, assuming that participants choose in line with expected utility theory, the observation that the most common switching point in financial lotteries was in line 4 indicates that most participants in our study could be classified as mildly risk averse.

Of course, the same framework of expected utility theory can be applied to health lotteries, where the ‘payoff’ is expressed not in monetary terms but in health outcomes. Participants will assign a utility to each day in perfect health. This again allows a decision-maker to compare the utility derived from a guaranteed number of healthy days to the expected utility derived as the probability weighted utility from the two possible numbers of healthy days under risk. Like in the case of financial risk, a risk-averse decision-maker under an expected utility framework would demand a premium of expected healthy days under risk compared with a certain number of healthy days. Our results indicate that, in the health domain, more decision-makers act in accordance with risk aversion than in financial lotteries. There was also a higher proportion of extreme choices always selecting the risky or guaranteed option.

Overall, not all participants acted in accordance with standard theories of expected utility as indicated by multiple switching or other inconsistent choice patterns. Such heterogeneity in switching behaviour, including participants who reversed their choices multiple times, suggests that considerations beyond expected utility, such as loss aversion, ambiguity preferences or different reference points, likely influence both financial and health-related risk decisions.

Finally, this study helped to highlight that talking to people about why they made certain decisions can elucidate their, potentially entirely rational, reasons for doing this, and in so doing explain decisions or decision patterns that appear irrational when viewed from an entirely theoretical perspective, or that vary significantly from the decision that another person (eg, a healthcare professional) may take in the situation. When quantitatively investigating risk tolerance in a similar way to this study, others have tackled the risk of unusual choice patterns by advising participants not to switch more than once.^26^ However, this approach was not taken in the current study, so as to minimise the potential influence of the researcher on participants’ choices, and to allow better determination of whether acutely unwell patients, and their carers, can engage in structured discussions of risk, which they proved able to do. Even without instructions regarding switching multiple times, the rate of ‘multiple switchers’ in the current study was slightly lower than in some previous studies which have used similar methods. In this study, 14.8% of participants switched multiple times in at least one lottery set, whereas in previous research this rate has been around 20%.^16^

### Limitations

The key limitations of this study may give rise to considerations for future work in this area. The ‘field notes’ which were recorded by the researcher, noting conversations they had with patient and carer participants, provided a level of insight into the rationale behind some of the apparently irrational choice patterns of participants, which may have applications in further understanding these behaviours. Examples of these for multiple switchers included making choices in questions in the financial lottery sets based on previous choices—for example, taking a ‘third time lucky’ approach (and thus choosing the risky option for every third question but guaranteed option for the others), or deliberately moderating overall risk by taking some riskier choices and then returning to the guaranteed options. A number of participants also made choices in the health lottery sets based on an assumption of either a specific symptom, or their current experience of ill health, which had led them to be present in the department, which could vary if they were asked the same questions at a different time. However, these field notes were collected informally, and future work may benefit from formalising the collection of reflections or comments from participants, to give greater insight into their rationale for the decisions they make. Additionally, despite the acute care setting, a relatively limited number of older people participated in this study, which may limit the reliability of the findings involving comparisons between people of different age groups. Similarly, although it was good that this study involved staff members from a number of different professions, some remained under-represented, such as nurses. This may be due in part to a difference in the role that these professionals play in making location of care decisions—doctors and physiotherapists may generally have a more direct impact on this decision at the front-door, but future work may consider recruiting those in other professions as well, including those working in extended scope or advanced practitioner roles, who may be involved in more independent location of care decision-making.

However, despite these limitations, a novel population was recruited to investigate their risk tolerance and preferences, and was able to meaningfully engage with the tasks. The inclusion of data from field notes, even informal ones, also provides insight and context for decisions that were made which has not been used in previous work of this type, in other contexts.

## Conclusion

Overall, this study demonstrated that staff, patients and carers from ambulatory care, front-door and/or HaH services are able to meaningfully engage with structured discussions about risk, and make decisions based on this information. It showed that risk tolerance for health-based decisions was generally lower than for financially-based decisions, among both patients currently experiencing an episode of ill health and staff and carers who may have had a range of current health states. Additionally, it showed that older people tended towards a more dichotomised approach to risk-taking in health-based decisions than younger people did, which may have direct implications for discussions of risks involved in various care locations, when these discussions occur at the hospital front-door.

Future work should consider formalising the collection of people’s rationale for risk-based decisions, and should seek to include participants representing a wide range of demographic characteristics. Further studies may also consider being conducted in other clinical settings, as well as explicitly evaluating risk tolerance for location of care.

The findings of this study may influence clinical practice by encouraging staff to engage in discussions of risk with patients, and carers, in a more structured or in-depth way than they previously have, as well as highlighting some of the patterns of difference in risk tolerance among certain groups, which may be entirely rational and may contribute to better communication between parties involved in location of care decision-making.

## Supplementary material

10.1136/bmjopen-2025-099032 online supplemental figure 1

10.1136/bmjopen-2025-099032 online supplemental figure 2

10.1136/bmjopen-2025-099032 online supplemental figure 3

## Data Availability

No data are available.
