# Supplementary material for: Novel assessment of risk tolerance in acute healthcare settings: a questionnaire-based study investigating risk tolerance of service users and staff in ambulatory care and front-door services
Source: BMJ Open. 2025 Nov 12;15(11):e099032. doi: 10.1136/bmjopen-2025-099032 (PMC12612725; doi:10.1136/bmjopen-2025-099032)
Supplement: online supplemental figure 1 [file bmjopen-15-11-s001.pdf]

Risk Preferences in Acute Medical settings

Thank you for agreeing to complete this questionnaire, which forms part of a research project being undertaken at the University of Birmingham by Ciara Harris.

Please be aware of the following:

- Completion of this questionnaire is **voluntary** – you do not have to complete it
- Your answers are **anonymous** – you cannot be identified from your answers
- Your answers are **confidential** – only the researcher will know what answers you give

If there is anything that you would like to ask, please feel free to ask the researcher at any time.

Section 1: Background

Please circle the correct response.

What gender do you identify as?

Male   Female   Other   Prefer not to say

Which age category are you in (years)?

16-20   21-30   31-40   41-50   51-60   61-64

65-74   75-84   85-94   95 and over

What is your ethnicity?

White:

British   Irish   Gypsy or Irish Traveller

Any other White background

Mixed / Multiple ethnic groups:

White and Black Caribbean White and Black African White and Asian

Any other Mixed / Multiple ethnic background

Asian / Asian British:

Indian Pakistani Bangladeshi Chinese

Any other Asian background

Black / African / Caribbean / Black British:

African Caribbean Any other Black / African / Caribbean background

Other ethnic group:

Arab Any other ethnic group

Prefer not to say

What type of service are you using?

Ambulatory Care Front-Door unit

Are you a patient or a carer (during this episode of care)?

Patient Carer

If patient: Would you be willing to be contacted regarding being interviewed for a second component of this study? If yes, please provide contact details (e.g. email address or telephone number)
