# Supplementary material for: Novel assessment of risk tolerance in acute healthcare settings: a questionnaire-based study investigating risk tolerance of service users and staff in ambulatory care and front-door services
Source: BMJ Open. 2025 Nov 12;15(11):e099032. doi: 10.1136/bmjopen-2025-099032 (PMC12612725; doi:10.1136/bmjopen-2025-099032)
Supplement: online supplemental figure 2 [file bmjopen-15-11-s002.pdf]

Risk Preferences in Acute Medical settings

Thank you for agreeing to complete this questionnaire, which forms part of a research project being undertaken at the University of Birmingham by Ciara Harris.

Please be aware of the following:

- Completion of this questionnaire is **voluntary** – you do not have to complete it
- Your answers are **anonymous** – you cannot be identified from your answers
- Your answers are **confidential** – only the researcher will know what answers you give

If there is anything that you would like to ask, please feel free to ask the researcher at any time.

Section 1: Background

Please answer the following questions

What gender do you identify as?

Male   Female   Other   Prefer not to say

What is your current job role?

Doctor (please indicate speciality):

Consultant   Registrar   Speciality Trainee   FY1/FY2

Therapist or Nurse:

Physio   OT   Nurse   Other (please specify):

Band 8 (or above)   Band 7   Band 6   Band 5

If Physio: Would you be willing to be contacted regarding being interviewed for a second component of this study? If yes, please provide contact details (e.g. email address or telephone number)

Number of years working since qualification?

15+ 11 - 14 6 - 10 2 - 5 Less than 2

Previous clinical experience:

(Please indicate how many years / months you have worked in Ambulatory Care or Front-Door services, and please indicate any other clinical areas in which you have worked for a significant period of time)

Type of service that you currently work in:

Ambulatory Care Front-Door Unit

[Ambulatory Care = service which replaces at least a portion of inpatient care episode, with a stay elsewhere, often in patient's own home (e.g. Hospital at Home, Early Supported Discharge, Ambulatory Care unit);

Front- Door Unit = short stay unit / area which patients are admitted to following unplanned presentation, where they receive care and are discharged in a short time period, often a maximum of 72 hours (e.g. acute medical unit, emergency department observation unit)]
