# Supplementary material for: Novel assessment of risk tolerance in acute healthcare settings: a questionnaire-based study investigating risk tolerance of service users and staff in ambulatory care and front-door services
Source: BMJ Open. 2025 Nov 12;15(11):e099032. doi: 10.1136/bmjopen-2025-099032 (PMC12612725; doi:10.1136/bmjopen-2025-099032)
Supplement: online supplemental figure 3 [file bmjopen-15-11-s003.pdf]

Section 2: Lotteries

Please follow the instructions below and answer the following 6 series' of questions.

For each one, remember that there are no right or wrong answers, it is your personal choices that we are interested in.

1) **Questions about financial risk, with ambiguity**

For each of the following questions, please select whether you would choose option A or option B, by selecting the relevant box. Option A guarantees £7 in each case; Option B has an unknown chance of a lower or higher amount, which differs each time.

For example, in question 1, option A guarantees you £7, while option B gives you an unknown chance of getting either £2 or £11 (indicated by ?%).

Question	Option A	Option B	Your Choice
1	Guaranteed £7	?% chance of £2 and ?% chance of £11	A or B<br>☐ ☐
2	Guaranteed £7	?% chance of £2 and ?% chance of £12	A or B<br>☐ ☐
3	Guaranteed £7	?% chance of £2 and ?% chance of £13	A or B<br>☐ ☐
4	Guaranteed £7	?% chance of £2 and ?% chance of £14	A or B<br>☐ ☐
5	Guaranteed £7	?% chance of £2 and ?% chance of £16	A or B<br>☐ ☐
6	Guaranteed £7	?% chance of £2 and ?% chance of £18	A or B<br>☐ ☐
7	Guaranteed £7	?% chance of £2 and ?% chance of £20	A or B<br>☐ ☐

2) Questions about financial risk, with ambiguity (higher payoffs)

For each of the following questions, please select whether you would choose option A or option B, by selecting the relevant box. Option A guarantees £70 in each case; Option B has an unknown chance of a lower or higher amount, which differs each time.

For example, in question 1, option A guarantees you £70, while option B gives you an unknown chance of getting either £20 or £110 (indicated by ?%).

Question	Option A	Option B	Your Choice
1	Guaranteed £70	?% chance of £20 and ?% chance of £110	A ☐ or B ☐
2	Guaranteed £70	?% chance of £20 and ?% chance of £120	A ☐ or B ☐
3	Guaranteed £70	?% chance of £20 and ?% chance of £130	A ☐ or B ☐
4	Guaranteed £70	?% chance of £20 and ?% chance of £140	A ☐ or B ☐
5	Guaranteed £70	?% chance of £20 and ?% chance of £160	A ☐ or B ☐
6	Guaranteed £70	?% chance of £20 and ?% chance of £180	A ☐ or B ☐
7	Guaranteed £70	?% chance of £20 and ?% chance of £200	A ☐ or B ☐

3) Questions about health risk, with ambiguity

Please imagine that you have a health condition which stops you from working / doing your normal daily activities. You have 2 treatment options – Treatment A guarantees full recovery for 70 days, Treatment B gives an unknown chance of 2 recovery periods, which vary in each question. For each of the following questions, please select whether you would choose option A or option B, by selecting the relevant box.

For example, in question 1, treatment A guarantees 70 days of full health, while treatment B gives you an unknown chance of either 20 days of full health or 110 days of full health (indicated by ?%).

When the treatment is finished, you will go back to how you are at the moment, regardless of which treatment option you choose, and there will be no further treatment.

Question	Treatment A	Treatment B	Your Choice
1	Guaranteed 70 days in full health	?% chance of 20 days in full health and<br>?% chance of 110 days in full health	A or B<br>☐ ☐
2	Guaranteed 70 days in full health	?% chance of 20 days in full health and<br>?% chance of 120 days in full health	A or B<br>☐ ☐
3	Guaranteed 70 days in full health	?% chance of 20 days in full health and<br>?% chance of 130 days in full health	A or B<br>☐ ☐
4	Guaranteed 70 days in full health	?% chance of 20 days in full health and<br>?% chance of 140 days in full health	A or B<br>☐ ☐
5	Guaranteed 70 days in full health	?% chance of 20 days in full health and<br>?% chance of 160 days in full health	A or B<br>☐ ☐
6	Guaranteed 70 days in full health	?% chance of 20 days in full health and<br>?% chance of 180 days in full health	A or B<br>☐ ☐
7	Guaranteed 70 days in full health	?% chance of 20 days in full health and<br>?% chance of 200 days in full health	A or B<br>☐ ☐

4) Questions related to financial risk

For each of the following questions, please select whether you would choose option A or option B, by selecting the relevant box. Option A guarantees £7 in each case; Option B gives a 50/50 chance of differing amounts each time.

For example, in question 1, option A guarantees you £7, while option B gives you a 50/50 chance of getting either £2 or £11.

Question	Option A	Option B	Your Choice
1	Guaranteed £7	50% chance of £2 and 50% chance of £11	A or B<br>☐ ☐
2	Guaranteed £7	50% chance of £2 and 50% chance of £12	A or B<br>☐ ☐
3	Guaranteed £7	50% chance of £2 and 50% chance of £13	A or B<br>☐ ☐
4	Guaranteed £7	50% chance of £2 and 50% chance of £14	A or B<br>☐ ☐
5	Guaranteed £7	50% chance of £2 and 50% chance of £16	A or B<br>☐ ☐
6	Guaranteed £7	50% chance of £2 and 50% chance of £18	A or B<br>☐ ☐
7	Guaranteed £7	50% chance of £2 and 50% chance of £20	A or B<br>☐ ☐

5) Questions related to financial risk (higher payoffs)

For each of the following questions, please select whether you would choose option A or option B, by selecting the relevant box. Option A guarantees £70 in each case; Option B gives a 50/50 chance of differing amounts each time.

For example, in question 1, option A guarantees you £70, while option B gives you a 50/50 chance of getting either £20 or £110.

Question	Option A	Option B	Your Choice
1	Guaranteed £70	50% chance of £20 and 50% chance of £110	A ☐ or B ☐
2	Guaranteed £70	50% chance of £20 and 50% chance of £120	A ☐ or B ☐
3	Guaranteed £70	50% chance of £20 and 50% chance of £130	A ☐ or B ☐
4	Guaranteed £70	50% chance of £20 and 50% chance of £140	A ☐ or B ☐
5	Guaranteed £70	50% chance of £20 and 50% chance of £160	A ☐ or B ☐
6	Guaranteed £70	50% chance of £20 and 50% chance of £180	A ☐ or B ☐
7	Guaranteed £70	50% chance of £20 and 50% chance of £200	A ☐ or B ☐

6) Questions related to health risk

Please imagine that you have a health condition which stops you from working / doing your normal daily activities. You have 2 treatment options – Treatment A guarantees full recovery for 70 days, Treatment B gives a 50/50 chance of 2 recovery periods, which vary in each question. For each of the following questions, please select whether you would choose option A or option B, by selecting the relevant box.

For example, in question 1, treatment A guarantees 70 days of full health, while treatment B gives you a 50/50 chance of either 20 days of full health or 110 days of full health.

When the treatment is finished, you will go back to how you are at the moment, regardless of which treatment option you choose, and there will be no further treatment.

Question	Treatment A	Treatment B	Your Choice
1	Guaranteed 70 days in full health	50% chance of 20 days in full health and 50% chance of 110 days in full health	A ☐ or B ☐
2	Guaranteed 70 days in full health	50% chance of 20 days in full health and 50% chance of 120 days in full health	A ☐ or B ☐
3	Guaranteed 70 days in full health	50% chance of 20 days in full health and 50% chance of 130 days in full health	A ☐ or B ☐
4	Guaranteed 70 days in full health	50% chance of 20 days in full health and 50% chance of 140 days in full health	A ☐ or B ☐
5	Guaranteed 70 days in full health	50% chance of 20 days in full health and 50% chance of 160 days in full health	A ☐ or B ☐
6	Guaranteed 70 days in full health	50% chance of 20 days in full health and 50% chance of 180 days in full health	A ☐ or B ☐
7	Guaranteed 70 days in full health	50% chance of 20 days in full health and 50% chance of 200 days in full health	A ☐ or B ☐
