# Supplementary material for: Novel assessment of risk tolerance in acute healthcare settings: a questionnaire-based study investigating risk tolerance of service users and staff in ambulatory care and front-door services
Source: BMJ Open. 2025 Nov 12;15(11):e099032. doi: 10.1136/bmjopen-2025-099032 (PMC12612725; doi:10.1136/bmjopen-2025-099032)
Supplement: Supplementary file 4 [file bmjopen-2025-099032.reviewer_comments.pdf]

PEER REVIEW HISTORY

BMJ Open publishes all reviews undertaken for accepted manuscripts. Reviewers are asked to complete a checklist review form and are provided with free text boxes to elaborate on their assessment. These free text comments are reproduced below.

ARTICLE DETAILS

Title (Provisional)

Novel assessment of risk tolerance in acute healthcare settings: a questionnaire-based study investigating risk tolerance of service users and staff in Ambulatory Care and front-door services

Authors

Harris, Ciara; Lohse, Johannes; Drouvelis, Michalis; Lasserson, Daniel S

VERSION 1 - REVIEW

**Reviewer**	**1**
**Name**	**Heo, Wookjae**
**Affiliation**	**Purdue University**
**Date**	**27-May-2025**
**COI**	**None**

Thank you for presenting an interesting study. However, the following issues need to be clarified explicitly:

1. The connection between financial risk tolerance and medical payments (or, health lotteries) is entirely missing. Financial risk tolerance or risk aversion primarily deals with input (e.g., investments) and the outcome (e.g., returns) associated with those initial input (e.g., investments). Risk aversion, foundational to risk tolerance, possesses several characteristics: losses are perceived twice as intensely compared to gains of equal utility; extremely high or low probabilities tend to be exaggerated; and probabilities on a continuum are often considered discretely by individuals. Such theoretical foundations are well-established. However, the current study's aim—to investigate health lotteries within medical settings—lacks a logical connection with financial risk tolerance or its underlying principle of risk aversion. It must be clearly explained why and how health lotteries relate logically to financial risk tolerance or risk aversion. Why do the authors need to investigate the association between financial risk tolerance and health lotteries? Logical explanation should be well introduced; not just "we wanted to do compare."

2. Additionally, in the last paragraph of the introduction and the methods section, the authors proposed studying financial risk tolerance among diverse groups such as healthcare

staff, patients, and carers. These groups have fundamentally different reward structures within the health lottery system. As mentioned above at the comment 1, risk aversion or financial risk tolerance is inherently based on empirical experiments involving inputs and corresponding rewards. Since the rewards corresponding to inputs differ significantly among the groups identified, the research objective becomes vague, appearing merely as an attempt to relate financial risk tolerance and health lotteries without clear logical grounding. Each group must be individually considered and analyzed, explicitly showing how they estimate inputs and associated rewards, thus demonstrating how financial risk tolerance operates distinctly within each group.

3. In addition to the comment 1 and 2, the deep logic should be clearly understood by examining basic utility functions related to risk aversion. Even a basic understanding of utility functions highlights that the exponential expression involving Euler's number (e) should at least be considered. Specifically, given that the authors' experimental framework originates from Tversky and Kahneman's experiments, the discussion must explicitly incorporate the economic utility function logic underlying risk aversion.

4. Moreover, the analysis presents only a simple distribution. What insights can we gain from distribution alone? Merely observing high or low values does not effectively explain the estimated relationships. At a minimum, demographic variables must be controlled to estimate accurately the relationship between financial risk tolerance and health lotteries. Simple observational distributions, as currently provided, offer no substantial statistical support.

**Reviewer**	**2**
**Name**	**Dibba, Yusupha**
**Affiliation**	**Partners In Health Sierra Leone, Clinical**
**Date**	**26-Aug-2025**
**COI**	**None**

Thank you for the opportunity to review the paper. This paper is indeed an interesting and novel approach to risk tolerance assessment, and I believe it will make a significant contribution to the current literature. I have a few comments that are meant to improve the paper.

1. There are a few typos/grammar issues that need to be looked at. e.g in page 6 line 32, page 14 line 18,

2. In the methods section, the study period and duration are not clearly defined. This is important. Hence, it is not clear whether this study was done prior to X taking over twitter or not.

3. It wasn't clear how the participant sample was gotten. "Limited previous research of this type in a healthcare setting meant that a sample size calculation was not possible, and target sample size was therefore based on the target number of participants in a previous outpatient-based study involving patients and doctors" - It is still important that it is clear how the sample size was reached in this particular study. - convenient sampling? etc,,

4. The data collection tool (Study questionnaire) was adopted. Was this a validated tool? if not, then it could be adapted from a similar study but customized and tested/validation.

5. Study questionnaire in Fig1 did not show any of the questions asked. It will be helpful to know if the questions asked are aligned with the description.

6. The online recruitment method of staff needs to be described in full detailed.

7. In the results, table 1b, the age intervals are not equal in all the categories. If there are reasons for that, it should be explained.

8. In the discussion section, the last paragraph (or the content) should be brought up before the paragraph with 'Finally ...'

VERSION 1 - AUTHOR RESPONSE

Reviewer: 1
Dr. Wookjae Heo, Purdue University
Comments to the Author:
Thank you for presenting an interesting study. However, the following issues need to be clarified explicitly:
1. The connection between financial risk tolerance and medical payments (or, health lotteries) is entirely missing. Financial risk tolerance or risk aversion primarily deals with input (e.g., investments) and the outcome (e.g., returns) associated with those initial input (e.g., investments). Risk aversion, foundational to risk tolerance, possesses several characteristics: losses are perceived twice as intensely compared to gains of equal utility; extremely high or low probabilities tend to be exaggerated; and probabilities on a continuum are often considered discretely by individuals. Such theoretical foundations are well-established. However, the current study's aim—to investigate health lotteries within medical settings—lacks a logical connection with financial risk tolerance or its underlying principle of risk aversion. It must be clearly explained why and how health lotteries relate logically to financial risk tolerance or risk aversion. Why do the authors need to investigate the association between financial risk tolerance and health lotteries? Logical explanation should be well introduced; not just "we wanted to do compare."

- Thank you for this suggestion. We agree that this contribution should be clarified. Risk aversion, as used in expected utility theory, can apply both to monetary and non-monetary outcomes. You are right to point out that most experimental methods for eliciting risk preferences have focussed on financial risk, but there is a smaller literature cited that focusses on health risks. We included both types of outcomes in our study and discuss whether the monetary stakes lotteries used in previous research capture similar levels of risk aversion than the health stakes lotteries we used. This question follows from a mixed picture in the existing literature on health lotteries.

We have added two paragraphs to the Introduction to explain the inclusion of both financial and health lotteries.

Most previous investigation of risk tolerance in behavioural economics has used financial choices as the basis for assessment of risk tolerance (e.g. [14-16]), and therefore this is the setting in which most risk elicitation methods have been validated. The basic finding from this literature and corresponding theories is that most people are moderately risk averse for the tasks and stakes used. However, there is some discussion in the literature to what degree risk preferences are domain specific [17].

In particular, there is some evidence that people treat financial and health-related risks differently [11 13]. For the purpose of this study, it is therefore logical to investigate both aspects of risk taking and their relationship, in order to determine if financial risk tolerance is a suitable proxy for health risk tolerance. Additionally, previous research involving patients has found that participants found it easier to understand health-related risk questions if they were presented with similar finance-related questions first [11], and therefore this approach was also taken in this study.

2. Additionally, in the last paragraph of the introduction and the methods section, the authors proposed studying financial risk tolerance among diverse groups such as healthcare staff, patients, and carers. These groups have fundamentally different reward structures within the health lottery system. As mentioned above at the comment 1, risk aversion or financial risk tolerance is inherently based on empirical experiments involving inputs and corresponding rewards. Since the rewards corresponding to inputs differ significantly among the groups identified, the research objective becomes vague, appearing merely as an attempt to relate financial risk tolerance and health lotteries without clear logical grounding. Each group must be individually considered and analyzed, explicitly showing how they estimate inputs and associated rewards, thus demonstrating how financial risk tolerance operates distinctly within each group.

- Thank you for this comment. As the lottery questions that were asked to each participant, in all groups, were identical, the rewards and risks associated with each choice were also identical between patient, carer and staff groups. We acknowledge that the different groups included in our study may differ in their demographic attributes and incentive structures when it comes to ambulatory admission decisions. The aim of this study is to introduce two elicitation methods for underlying risk preferences that inform such decisions and whether underlying preferences match across groups and task formats.

- As you suggest, we analyse both groups separately in multiple regression models. In both groups we found a significant difference between health lotteries and financial lotteries, that is slightly less pronounced for health care workers. One reason could be, that in the health-related lottery questions, it is of course possible that healthcare staff were more sceptical of the concept of a hypothetical treatment that could provide full symptomatic relief for a defined period of time, but as the questions still related to which treatment they would choose for themselves, it may be expected that their range of motivations for these choices do not meaningfully differ from the range of motivations in the non-healthcare-staff population.

3. In addition to the comment 1 and 2, the deep logic should be clearly understood by examining basic utility functions related to risk aversion. Even a basic understanding of utility functions highlights that the exponential expression involving Euler's number (e) should at least be considered. Specifically, given that the authors' experimental framework originates from Tversky and Kahneman's experiments, the discussion must explicitly incorporate the economic utility function logic underlying risk aversion.

- Thank you for this suggestion, paragraphs have been added to the Discussion. In particular, this discussion highlights how our experimental data are linked to basic expected utility theory and how expected utility theory applies to both financial and health related outcomes. We also highlight in this discussion how some observed choice patterns cannot be explained easily by EUT and may require alterative theoretical explanations.

- As per your suggestion we also include a more technical paragraph relating how a standard exponential utility function could be used in modelling choices, although we feel that his may be overly technical for more applied readers.

Previous studies employing lottery-style questions to elicit risk tolerance, most often in financial contexts, have focused on decision makers' aversion to monetary risk. Within the commonly employed framework of expected utility theory, the decision maker evaluates each option based on its expected utility i.e. the probability-weighted average of the utility derived from all possible monetary payoffs under each option chosen. A commonly assumed functional form is the exponential utility function, $U(x) = \frac{1 - e^{-rx}}{r}$*,, where x denotes the outcome (e.g., monetary payments or number of days in full health), and r>0 captures the degree of risk aversion. For a risk-neutral decision maker (r=0), the utility function is assumed to be linear (U(x)=x), meaning choices depend only on expected values. Risk aversion implies r > 0. Rational agents, under this model, should always select the option with the higher expected utility. Accordingly, in the current study, risk neutral participants would be expected to switch from the certain to the risky option once the expected value of the latter exceeds that of the former (e.g., at line 3 in the questions with known probabilities). Similarly, risk aversion demands a premium in expected value for choosing a risky outcome, while risk loving decision makers would even pick a risky option if it offers a lower expected value than the corresponding guaranteed option. Accordingly, assuming that participants choose in line with expected utility theory, the observation that the most common switching point in financial lotteries was in line 4, indicates that most participants in our study could be classified as mildly risk averse.*

Of course, the same framework of expected utility theory can be applied to health lotteries, where the "payoff" is expressed not in monetary terms but in health outcomes. Participants will assign a utility to each day in perfect health. This again allows a decision maker to compare the utility derived from a guaranteed number of healthy days to the expected utility derived as the probability weighted utility from the two possible numbers of healthy days under risk. Like in the case of financial risk, a risk averse decision maker under an expected utility framework would demand a premium of expected healthy days under risk compared to a certain number of healthy days. Our results indicate that, in the health domain, more

decision makers act in accordance with risk aversion than in financial lotteries. There was also a higher proportion of extreme choices always selecting the risky or guaranteed option.

Overall, not all participants acted in accordance with standard theories of expected utility as indicated by multiple switching or other inconsistent choice patterns. Such heterogeneity in switching behaviour, including participants who reversed their choices multiple times, suggests that considerations beyond expected utility, such as loss aversion, ambiguity preferences, or different reference points, likely influence both financial and health-related risk decisions.

4. Moreover, the analysis presents only a simple distribution. What insights can we gain from distribution alone? Merely observing high or low values does not effectively explain the estimated relationships. At a minimum, demographic variables must be controlled to estimate accurately the relationship between financial risk tolerance and health lotteries. Simple observational distributions, as currently provided, offer no substantial statistical support.

- Thank you for this comment. Further analyses were conducted to compare patterns of risk tolerance between groups with different demographic characteristics (e.g. ethnicity, profession, length of professional experience, staff vs patients & carers), but are not presented in this manuscript, in which our aim is to present the key findings of the study. Our aim in presenting distributions, as opposed to an individual statistical representation of the risk tolerance of different groups is, in part, to demonstrate the range of risk tolerance levels within the populations – if this were not done, it would obscure the findings of how these tolerance levels are distributed and risk mis-representing whole groups as having homogeneous approaches to risk. Additionally, multiple regression results and odds ratios (with 95% confidence intervals and p values) are reported for comparisons between lottery types, separated into staff and patient & carer groups (*"multiple regression, with all lottery sets included, identified statistically significant slope coefficients for lottery type among healthcare professionals (-0·492, 95% CI -0·971 to -0·013, p = 0·044) and among patients & carers (-0·612, 95% CI -0·922 to -0·302, p <·001) … Both staff and patients & carers had statistically significant odds of having a higher switch point in the health lottery sets than the financial ones (Staff: OR = 1·576; 95% CI, 1·167 to 2·128; p = ·003. Patients & carers: OR = 1·613; 95% CI, 1·303 to 1·996; p < ·001),"*); and the p value is reported for the comparison between older and younger people's choice distributions (*"When comparisons were made using the number of times the guaranteed option was chosen, the distributions of these showed a statistically significant difference (p = 0·002) between older and younger people"*).

Reviewer: 2
Dr. Yusupha Dibba, Partners In Health Sierra Leone
Comments to the Author:
Thank you for the opportunity to review the paper. This paper is indeed an interesting and novel approach to risk tolerance assessment, and I believe it will make a significant contribution to the current literature. I have a few comments that are meant to improve the paper.

1. There are a few typos/grammar issues that need to be looked at. e.g in page 6 line 32, page 14 line 18,

- Thank you for highlighting this, we have more closely re-read the manuscript to identify any remaining errors. Should any further issues remain, we would be happy to correct them.

2. In the methods section, the study period and duration are not clearly defined. This is important. Hence, it is not clear whether this study was done prior to X taking over twitter or not.

- Thank you for this suggestion, the recruitment periods for both groups have been added in the Methods section.

  Participants were also encouraged to share information about the study with colleagues, and those working in the department where patients and carers were recruited were invited to participate in-person. *Staff were recruited between August and December 2020*. Patients and carers were recruited in-person, while they were waiting in an Ambulatory Care unit of a busy city hospital in Birmingham, UK*, between September and December 2020*.

3. It wasn't clear how the participant sample was gotten. "Limited previous research of this type in a healthcare setting meant that a sample size calculation was not possible, and target sample size was therefore based on the target number of participants in a previous outpatient-based study involving patients and doctors" - It is still important that it is clear how the sample size was reached in this particular study. - convenient sampling? etc,,

- Thank you for this suggestion, further information about the sampling method has been added to the Methods section.

  Limited previous research of this type in a healthcare setting meant that a*n ex-ante* sample size calculation *based on statistical-power considerations* was not possible, and target sample size was therefore based on the target number of participants in a previous outpatient-based study involving patients and doctors [12]. *A convenience sampling approach was used for patients and carers, thorough inviting those who attended the Ambulatory Care unit during the study period. A combination of convenience and snowball sampling was used for staff participants, through approaching relevant individuals through professional societies and social media, where eligible individuals were likely to see the invitation, and encouraging participants to share information about the study with colleagues.*

4. The data collection tool (Study questionnaire) was adopted. Was this a validated tool? if not, then it could be adapted from a similar study but customized and tested/validation.

- Thank you for highlighting this. The questionnaire was adapted from previous research, and the following sentence has been added to explain this.

  The questionnaire was adapted from previous research which developed and validated a variety of tools to assess aspects of financial risk preferences [16], and adaptations were made for this study based on pilot feedback, to increase ease of understanding.

5. Study questionnaire in Fig1 did not show any of the questions asked. It will be helpful to know if the questions asked are aligned with the description.

- Figure 1 is an example of one of the lottery sets, including the seven ‘lottery questions’, which are in the table. Participants selected box ‘A’ or ‘B’ in the ‘Your Choice’ column to answer each question. The full questionnaire is now also included as a supplementary file.

6. The online recruitment method of staff needs to be described in full detailed.

- Thank you for this suggestion, further information about the staff recruitment has been added to the Methods section.

  Staff participants were primarily recruited online, via routes such as Twitter and emails from relevant professional groups. *Emails and social media posts included brief information about the study and eligibility criteria, alongside access to a full participant information sheet and a link to participate in the study. Participants were also encouraged to share information about the study with colleagues, and those working in the department where patients and carers were recruited were invited to participate in-person. Staff were recruited between August and December 2020.* Patients and carers were recruited in-person,

7. In the results, table 1b, the age intervals are not equal in all the categories. If there are reasons for that, it should be explained.

- Thank you for this suggestion, an explanation for this is now included in the first Results paragraph.

  Patient & carer participants were aged from 16 to over 85 years old, and had a range of ethnicities (Table 1b). *An additional cut-off point of age categorisation was added at 64 years, in order to allow analyses based on those aged less than 65 years, and those aged 65 years and older, in line with common practice of categorising 65 years as the minimum age for ‘older people’* [20].

8. In the discussion section, the last paragraph (or the content) should be brought up before the paragraph with 'Finally ...'

- Thank you for highlighting this issue, the previous final discussion paragraph has been moved above the one beginning ‘Finally,…’, as you suggested.

VERSION 2 - REVIEW

**Reviewer**	**1**
**Name**	**Heo, Wookjae**
**Affiliation**	**Purdue University**
**Date**	**19-Oct-2025**
**COI**	

Thank you for adopting the comments well in the manuscript.
